# Efficacy of mobile health interventions in the conservative management of chronic low back pain in low- and middle-income countries: a systematic review, meta-analysis, and trial sequential analysis

**DOI:** 10.1097/PR9.0000000000001242

**Published:** 2025-02-13

**Authors:** Babina Rani, Mayank Gupta, Venkata Ganesh, Rajni Sharma, Anuj Bhatia, Babita Ghai

**Affiliations:** a Department of Physical and Rehabilitation Medicine, Post Graduate Institute of Medical Education and Research, Chandigarh, India; b Department of Anaesthesiology, All India Institute of Medical Sciences, Bathinda, Punjab, India; c Department of Anaesthesia and Intensive Care, Post Graduate Institute of Medical Education and Research, Chandigarh, India; d Department of Paediatrics, Post Graduate Institute of Medical Education and Research, Chandigarh, India; e Department of Anaesthesia and Pain Medicine, University of Toronto Health Policy Management and Evaluation, Toronto, ON, Canada; f Director of the Comprehensive Integrated Pain Program at University Health Network, Toronto, ON, Canada

**Keywords:** Chronic low back pain, Mobile health, mHealth, Low- and middle-income countries

## Abstract

Chronic low back pain (CLBP) is a major global health issue, particularly severe in low- and middle-income countries (LMICs), where health care resources and accessibility are limited. Mobile health (mHealth) interventions offer a promising solution by leveraging technology to deliver health care services remotely. This review aims to evaluate the effectiveness of mHealth interventions in managing CLBP in LMICs. A comprehensive search of electronic databases was performed for studies published until June 2024, evaluating mHealth interventions for CLBP in LMICs. Primary outcomes measured were pain intensity and disability, while secondary outcomes included quality of life (QoL). Risk of bias was assessed using Cochrane risk-of-bias tool (RoB2), and quality of evidence was evaluated using GRADE. Robustness of meta-analysis results was assessed via trial sequential analysis (TSA). Seven studies met the inclusion criteria. The mHealth interventions significantly reduced the overall pain intensity (MD = −1.11, 95% CI: −1.75, −0.46) and disability (MD = −6.59, 95% CI: −10.65, −2.54). Subgroup analysis indicated greater effectiveness of short-term interventions (<6 weeks) in reducing pain and Oswestry disability index (ODI) vs long-term interventions (>6 weeks). mHealth interventions notably reduced pain and ODI scores vs unsupervised programs but showed no significant difference compared to in-person programs. The z-score line remained within TSA boundaries. mHealth interventions show potential in reducing pain and disability among patients with CLBP in LMICs, although with inconclusive impact on QoL. The high heterogeneity and limited number of studies underscore the need for further research with greater sample size to validate these findings and explore the long-term benefits and implementation challenges of mHealth in resource-constrained settings.

## Abstract 2

Supplemental Digital Content is Available in the Text.

Mobile health interventions effectively reduce pain and disability in patients with chronic low back pain in low- and middle-income countries, especially compared to unsupervised care, but their impact on quality of life remains uncertain.

## 1. Introduction

Chronic low back pain (CLBP) poses a significant global health challenge, affecting the quality of life of the suffering individuals. The Global Burden of Disease Study 2021 identified low back pain (LBP) as the leading cause of years lived with disability (YLDs) worldwide, affecting 619 million individuals, with a projected rise of 36.4% by 2050.^17^ As highlighted in this study, the challenges in managing LBP differ markedly between low-and middle-income countries (LMICs) and high-income countries (HICs), reflecting disparities in health care resources, infrastructure, and socioeconomic contexts. According to the World Bank classification in 2023, countries with a gross national income (GNI) per capita of less than $1,145 are considered low-income, those between $1,146 and $4,515 are classified as lower middle-income, those with between $4,516 and $14,005 are deemed upper middle-income, and those with a GNI per capita exceeding $14,005 are categorized as high-income countries.^2^

The biopsychosocial impact of LBP is substantial, particularly among the working-age population,^17^ and more so in the developing nations.^4,17^ Health care utilization rates for LBP vary globally, ranging from 67% in the United States to 48% in Europe, with a considerable proportion of LBP patients failing to seek proper care.^1^ However, the existing evidence originates predominantly from the developed countries, thus limiting its generalizability to LMICs. Despite the clinical guidelines encouraging the use of conservative approaches (high value) as first-line management, there have been reports of overutilization of health care services for LBP, especially the interventional procedures (low-value), more so in the developed nations.^25,40^ Recent evidence suggests that hospitalization rates due to LBP in LMICs ranges between 13.4% and 18.7%,^14^ as against 3.2% for HICs.^15^

Comprehensive CLBP care involves self-care practices, educational initiatives, various nonpharmacological therapies (high value care), and a combination of pharmacological and interventional therapies (low value care) including the use of opioids. By empowering individuals through education and self-care as the first-line treatment, evidence-based guidelines endeavour to enhance the overall management and quality of life of individuals dealing with LBP.^42,46^ However, a significant global disparity exists between research-based best practices and actual clinical implementation of high-value care for CLBP, particularly in LMICs.^18^ This gap is largely driven by overwhelming patient volumes, insufficient health care provider: patient ratios, remote and rural location of patients hence difficult accessibility to health care systems in cities, and the time-intensive nature of evidence-based interventions.

While advancements in health care have led to various interventions, the accessibility and affordability of effective treatments remain a critical concern, particularly in LMICs. Recognizing the potential of technology to bridge this gap, mobile health (mHealth) interventions have emerged as a promising avenue for delivering health care services. The mHealth has been defined by WHO Global Observatory for eHealth as “medical and public health practice supported by mobile devices, such as mobile phones, patient monitoring devices, personal digital assistants, and other wireless devices.”^12^ Although the widespread adoption of mobile devices consequent to a surge in technology has improved the health care delivery in the developed nations,^6^ implementing mHealth interventions in developing nations poses a significant challenge due to the limited smartphone penetration, digital literacy, unreliable internet connectivity and accessibility, and/or the unaffordability of data plans. The economic constraint may hinder the widespread adoption and effectiveness of such interventions in these regions.

The utilization of smartphones and other mobile devices for health-related purposes has expanded rapidly, offering opportunities to deliver timely and personalized interventions to individuals with CLBP, particularly in developed nations.^21,31^ However, the existing literature lacks a comprehensive synthesis of the evidence regarding the efficacy of mHealth interventions in addressing CLBP in LMICs. This systematic review and meta-analysis aimed to comprehensively review and synthesize existing research on the effectiveness of mHealth interventions in managing CLBP in LMICs and to evaluate their impact quantitatively through a meta-analysis. The insights derived from this study will contribute to the development of targeted strategies, ensuring that innovative technologies are harnessed effectively to address the complex and pressing issue of CLBP in resource-constrained settings.

## 2. Methods

This review was conducted in accordance with the Preferred Reporting Items for Systematic Reviews and Meta-Analysis (PRISMA) guidelines.^34^ The review protocol was registered on PROSPERO (CRD42024528445), an international prospective register of systemic reviews.

### 2.1. Data sources and search strategy

The electronic databases (Medline via PubMed, Scopus, Web of Science, EMBASE, CINAHL via EBSCOhost) were systematically searched for the available literature on effectiveness of mHealth interventions in reducing pain and disability in subjects with CLBP. The literature search included studies (in English) published till June 2024. In addition, hand-searching was done through the reference lists of the included studies. The search strategy consisted of keywords and MeSH related to mHealth intervention (“mobile health,” “smartphone,” “telehealth,” “telemedicine,” “telerehabilitation,” “app-based,” “text message,” “virtual”) and chronic low back pain (“low back pain,” “chronic low back pain,” “non-specific low back pain,” “lumbar radiculopathy”). The search strategies as they have been run are listed in Supplementary File 1, http://links.lww.com/PR9/A281.

### 2.2. Eligibility criteria

The eligibility criteria for studies to be included in this review were defined as per the research question—“Are mHealth interventions for chronic low back pain in low- and middle-income country setting effective in reducing pain and related disability?” We used a PICOS (Population, Intervention, Comparator, Outcome, Study design) approach to identify studies for inclusion in this review.

#### 2.2.1. Population

Studies of human participants of age 18 years or more suffering from nonspecific chronic low back pain (more than 3 months duration) not requiring surgical intervention in the LMIC setting were eligible. The studies on subjects with organic, metabolic, traumatic, and/or surgical cause of CLBP were excluded from the review.

#### 2.2.2. Intervention

The intervention of interest was digital or mobile health (mHealth) intervention accessible via smartphone/tablet (including smartphone application/web-based/videoconferencing/video-based) aimed at reducing pain and disability in patients with CLBP. The interventions delivered to the patients in LMICs only were studied in this review. Studies focussing on wearable devices/activity trackers were not included. Studies on the use of virtual reality to manage CLBP were not included in this review.

#### 2.2.3. Comparator

The mHealth intervention was compared against an active control group receiving any other form of nondigital intervention (in-person management or unsupervised management or any other form of education-based treatment) or an inactive control group receiving no treatment.

#### 2.2.4. Outcome

The primary outcome of interest included measurement of pain and related disability in CLBP subjects. Any mHealth study reporting the measurement of these outcomes with validated instruments/scales were included. Only trials where validated measures for pain and function were evaluated a minimum of 2 times throughout the study duration were taken into consideration. The secondary outcomes included quality of life, measured with validated instruments/scales such as Euro QoL, Short form-6, 12, or 36, etc.

#### 2.2.5. Study design

Any experimental-interventional design (randomized or nonrandomized), clinical/cohort trials that investigate the effects of any form of mobile health intervention against a comparator in CLBP management were considered for the review. Nonempirical research, opinion pieces, and conference abstracts were excluded. Systematic reviews and other evidence synthesis were not included. However, reference lists of relevant systematic reviews were manually searched to identify any additional article. We did not search grey literature.

Table 1 describes the eligibility criteria in PICOS format.

**Table 1:** Eligibility criteria in population, intervention, comparator, outcome, study format.

	Inclusion	Exclusion
Patient population	CLBP > 12 wk, both genders Subjects from LMIC Nonspecific CLBP	CLBP of any organic/systemic/metabolic/surgical/traumatic cause
Intervention	Digital or mobile health (mHealth) intervention accessible via smartphone/tablet (including smartphone application/web-based/videoconferencing/video-based)	Studies focussing on wearable devices/activity trackers Studies on the use of virtual reality to manage CLBP
Comparator	An active control group receiving any other form of nondigital intervention (in-person management or unsupervised management or any other form of education-based treatment), or An inactive control group receiving no treatment or in waitlist	
Outcome measures	Pain (VAS, NRS, NPRS) Function (RMDQ, ODI, MODI, QBPDS) Outcomes evaluated a minimum of 2 times throughout the study duration	
Study design	RCT, nonrandomized trials, cohort (English full-text)	Case report, case series, reviews, cross-sectional studies Poster presentations, conference proceedings, editorials

CLBP, chronic low back pain; LMIC, low- and middle-income countries; MODI, modified Oswestry disability index; NPRS, numeric pain rating scale; NRS, numeric rating scale; ODI, Oswestry disability index; QBPDS, Quebec back pain disability scale; RCT, randomized controlled trial; RMDQ, Roland Morris disability questionnaire; VAS, visual analog scale.

### 2.3. Screening and data extraction

The search results from the electronic databases were then imported into Rayyan platform, which facilitates collaboration and discussion among reviewers and allows to save the full-text version of potentially relevant studies for further screening. After duplicate removal, 2 independent reviewers (B.R., M.G.) then performed title followed by abstract screening, for possible inclusion in the review as per the eligibility criteria. Potentially eligible records and records where eligibility could not be ascertained from abstracts were included for full-text screening. Any disagreements were resolved through a mutual discussion or with the help of third reviewer (V.G.) where necessary. A final list of the included studies was created in a reference manager software (EndNote 21).

From each included study, the following data were extracted using a structured table:

(1) Bibliographic characteristics, including first author, citation, year, and country.
(2) Study design.
(3) Subject information including sample size, age (mean/median with measures of dispersion), inclusion criteria, population characteristics.
(4) Details of intervention in all groups, including type of management, along with number, duration and frequency of sessions, total treatment duration.
(5) Outcome measures for variables of interest, and
(6) Results.

Corresponding authors of included articles were contacted if any information was missing from the publication. Sample data sheets were created and pilot tested for entering extracted data.

### 2.4. Risk of bias assessment

Two authors (B.R., M.G.) independently assessed the quality of the included studies, using Version 2 of the Cochrane risk-of-bias tool for randomized trials (RoB 2). The RoB 2 tool consists of 5 domains that evaluate bias arising from: the randomization process, deviations from intended interventions, missing outcome data, measurement of outcome, and selection of reported results. Overall risk-of-bias is judged as high, low, or some concerns.^41^ In case of any disagreements in any of the domain, a third author (B.G.) rated that specific domain.

### 2.5. Quality of evidence

To assess the quality of evidence of each outcome evaluated in this SRMA, the Grading of Recommendations Assessment, Development and Evaluations (GRADE) method was used.^37^ The evaluation criteria included the following dimensions: risk of bias, inconsistency, indirectness, imprecision, and publication bias. The scoring of the certainty of evidence for each particular outcome was done as “High,” “Moderate,” “Low,” or “Very low.”

With an initial a-priori ranking of “high” certainty to randomized controlled trials (RCTs), each study was then downgraded or upgraded depending on the identifiable bias. The various reasons to downgrade in different dimensions are described elsewhere.^19^

The certainty of evidence was rated up if (1) the effect size was large (and there is no substantial imprecision, with small effect sizes also being plausible); (2) a dose–response relationship was present; and (3) when all plausible confounders would reduce a demonstrated effect or suggest a spurious effect when the effect shown was neutral.^19^

### 2.6. Trial sequential analysis

To ensure the reliability and robustness of the results of our meta-analysis, we employed a trial sequential analysis (TSA) approach. This approach accounts for multiplicity due to repeated significance testing, and it can establish when firm evidence is reached in a cumulative meta-analysis.^9^ This is done by including the studies 1 by 1 into the meta-analysis and by considering each analysis in a manner similar to an interim analysis in a RCT, and accounting for the type I and II errors accordingly. Trial sequential analysis calculates a required information size; this is defined as the number of events or patients required to detect or reject an assumed intervention effect in a meta-analysis. In other words, it estimates when the effect is large enough to be unaffected by further studies, and it can be performed on each main outcome. If the required information size is not achieved, trial sequential analysis adds uncertainty to the estimate with less uncertainty being added as the sample size grows closer to the required information size.^44^ This results in a lower threshold of statistical significance than the usual 5% (*P* = 0.05) when the required information size has not been reached.

For this meta-analysis, we estimated the required sample size for the calculated effect size for the intervention, considering a type I error of 5% and a power of 80% (ie, type II error of 20%). Using TSA viewer (version 0.9.5.10-Beta, Copenhagen Trial Unit, Centre for Clinical Intervention Research, Denmark), the TSA was conducted for primary outcomes to determine the likely accuracy of the results of the meta-analysis using O'Brien-Fleming alpha spending method, by calculating the probability of the result being correct, taking into account the number of studies and participants involved. The TSA assists in calculating the required information size (RIS), thereby quantifying the statistical reliability of our data in the cumulative meta-analysis while adjusting the significance levels sparse data.^45^

By applying TSA, we assessed whether the cumulative evidence is sufficiently powered to confirm the effect of mHealth interventions on CLBP in LMICs. This involved plotting the cumulative z-curve and RIS boundaries, evaluating if the existing studies collectively reach the necessary information size to draw conclusive evidence, or if further studies are warranted.

### 2.7. Statistical analysis

The summative and subgroup data, as well as heterogeneity assessments, were generated using the Cochrane platform RevMan Web (https://revman.cochrane.org/info). To estimate the pooled effects for a comparison between mHealth and control groups, the changes in primary and secondary outcomes were analyzed using mean difference (MD) with 95% confidence intervals (CI), depending on the scale of measurement used in the included studies. In case of multiple follow-ups for any outcome in the included studies, the value for the last follow-up was used for analysis. For the studies that did not mention the mean change in outcome with either change in SD or with 95% CI, the calculation of change in SD was done using the formula^5^:

$${\text{SD}}_{\text{change}}=\sqrt{SD_{\text{baseline}}^{2}}+SD_{\text{final}}^{2}\left(2\times r\times {\text{SD}}_{\text{baseline}}\times {\text{SD}}_{\text{final}}\right)$$

Where r = correlation coefficient (if not mentioned, r is assumed to be 0.7).

#### 2.7.1. Meta-analysis

A random-effects model was employed to pool the effect sizes, considering the expected variability among the included studies due to differences in intervention types, populations, and settings. For outcomes with insufficient data or significant heterogeneity, a narrative synthesis approach was applied. Subgroup analyses were performed to explore potential effect modifiers, such as the type of mobile health intervention (eg, mobile apps, tele-based interventions), the duration of the intervention (eg, lesser or greater than 6 weeks), and the type of control intervention (in-person supervised vs unsupervised).

#### 2.7.2. Heterogeneity assessment

Statistical heterogeneity among the studies was assessed using the I^2^ statistic and χ^2^ test. An I^2^ value greater than 50% was considered indicative of substantial heterogeneity. A sensitivity analysis was conducted by excluding the studies with a high heterogeneity to assess the robustness of the overall results. The exclusion was performed using sequential and combinatorial algorithms.^35^

## 3. Results

### 3.1. Study selection and characteristics

**Figure 1.**
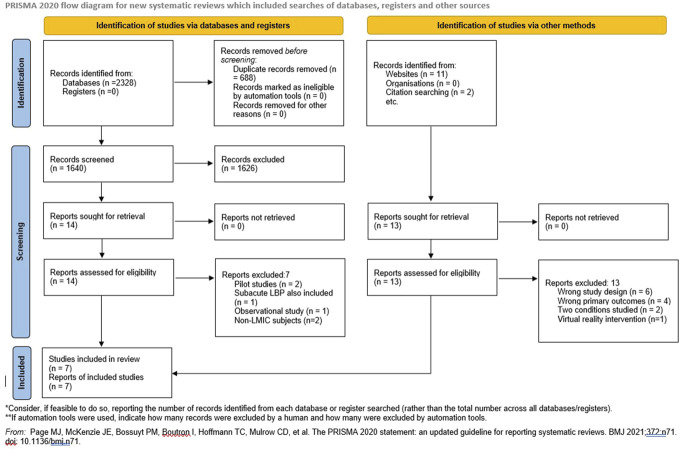
Preferred reporting items for systematic reviews and meta-analyses (PRISMA) flowchart.

A comprehensive literature search identified 2,341 studies, of which 7 studies evaluating the mHealth interventions in CLBP subjects met the eligibility criteria for this review, as illustrated in the PRISMA flowchart (Fig. 1). No nonrandomized study was found eligible for inclusion. The included studies varied in sample size, ranging from 41 to 93 participants. The study population consisted of adults with CLBP, with a mean age of 46.04 ± 10.47 years in the mHealth intervention group and 45.78 ± 11.46 years in the control group. Gender information was available in 5 of the 7 studies, totalling 136 females and 113 males. The average pain duration, reported in 5 out of 7 studies, was 13.65 months for the mHealth group and 12.93 months for the control group. The included mHealth intervention studies were conducted in LMICs, specifically Turkey (n = 3),^24,32,33^ Nigeria (n = 2),^13,29^ India (n = 1),^8^ and Jordan (n = 1).^1^

The studies included in the analysis utilized various types of mobile technologies to deliver interventions to the experimental group. Four out of 7 studies used Android-based mobile applications, while the remaining 3 employed videoconferencing platforms for telemedicine. None of the studies used any form of web-based intervention. The intervention duration ranged from 4 to 12 weeks.

The demographics, the specific characteristics, and mHealth intervention features of each included study are listed in Tables 2–4, respectively.

**Table 2:** Demographics of included studies' participants.

	Age EG (y)		Age CG (y)		Sample size		Overall gender distribution		Average pain duration EG		Average pain duration CG	
	Mean	SD	Mean	SD	EG	CG	F	M	Mean	SD	Mean	SD
Almhdawi 2020^2^	40.48	7.22	41.7	6.35	21	20	21	20	NR	NR	NR	NR
Fatoye 2020^13^	47.3	11.6	50	10.7	24	32	NR	NR	9.8 m	2.7	8.3 m	3.2
Karaduman 2023^24^	46.31	12.34	46.5	12.73	22	22	30	36	21.27 w	4.34	20.72 w	3.67
			43.95	17.06		22					21.65 w	5.03
Mbada 2019^29^	47.3	11.6	50	10.7	21	26	34	13	9.76 m	2.7	8.31 m	3.2
Okudan 2023^32^	52.57	5.03	50.8	4.69	15	15	21	24	NR	NR	NR	NR
	52.93	4.94			15							
Ozden 2022^33^	40.1	1.6	42.3	1.6	25	25	30	20	20.6 m	26.9	22.4 m	29.8
Chhabra 2018^8^	41.4	14.2	41	14.1	45	48	NR	NR	22.8 m	22	28 m	25.5

CG, control group; EG, experimental group; F, female; M, male; m, months; NR, not reported; w, weeks.

**Table 3:** Data extraction of included studies.

Author	Country	Study design	Patient	Treatment duration, frequency	Intervention	Control	Outcome	Outcome assessment	Results
Chhabra et al. 2018^8^	India	Single-blinded RCT	Mechanical CLBP >12 wk, NRS > 5 Age > 18	12 wk	App- snapcare + written prescription	Written prescription (medicine, physical activity including HEP)	NPRS, MODI App group only: Daily physical activity, CSS	Baseline, 12 wk	EG, CG: significant improvement in pain and disability (*P* < 0.05) EG: Significantly greater decline in disability (*P* < 0.001)
Mbada et al. 2019^29^	Nigeria	Quasi-experimental	CLBP with directional preference for McKenzie extension protocol Age = 20–65	8 wk 3/wk	App- (TBMT + education)	CBMT + educational instructions	VAS, ODI, RMDQ, SF12	Baseline, 4, 8 wk	Mobile-app platform of the McKenzie extension protocol has comparable clinical outcomes with the traditional clinic-based McKenzie therapy
Fatoye et al. 2020^13^	Nigeria	RCT	CLBP with directional preference for McKenzie extension protocol Age = 20–65	8 wk 3/wk	App- (TBMT + education)	CBMT + educational instructions	ODI, SF6, CUA (QALY), ICER	Baseline, 4, 8 wk	ODI: No significant between-group difference at week 4, 8
Almhdawi et al. 2020^2^	Jordan	Double-blinded RCT	CLBP > 3 m, VAS > 3 N = 41, office workers Age = 30–55	6 wk	App- relieve my back (evidence-based instructions + exc + 4 phone notifications)	Placebo version of app (instruction about nutrition + phone notifications)	VAS, ODI, SF12, SQI, IPAQ, “Google play” firebase logs	Baseline, 6 wk	Improvement in pain, disability, and QoL- more in EG than CG
Ozden 2022^33^	Turkey	Double-blind, 2-armed RCT	CLBP of >3 mo Age: 18–65	8 wk Once/day 10 reps each	Video exercise-based telerehab software (Fizyoweb)	Paper-based conventional rehab	VAS, ODI, SF36, TSK, TUG, FTST, EARS	Baseline, 8 wk	Improvement in pain, function, QoL, kinesiophobia, satisfaction, and motivation - more in EG than CG
Okudan 2023^32^	Turkey	Single-blinded RCT	CLBP > 12 wk caused by facet joint arthrosis (level 1 and 2) Age = 40–65	6 wk 2/wk 45 min each session 8 reps–2 sets–12 reps–3 sets	Telerehabilitation EG 1 = exercise EG2 = exercise + PNE	(On waiting list) maintain daily routine	NPRS at rest, activity, ODI, PBQ, SF-12v2, GROC	Baseline, 6 wk	Significant group-by-time interaction for NPRS, ODI, and organic pain belief EG2 showed better improvement in only organic pain belief
Karaduman 2023^24^	Turkey	Randomized, single-blind trial design	CLBP with RMDQ > 4, and fear of movement Age > 18	4 wk (12 sessions) 20–30 min exc program, 3/wk	TS group: Stabilisation exercise protocol online via videoconference, under PT guidance	IPS group: Stabilisation exercises in the hospital, under PT guidance US group: Stabilisation exercise protocol as HEP, comprehensively explained through handout + telephone calls from PT at end of week 1, 2, 3	NRS, ODI, TSK	Baseline, 4 wk	Tele-supervised stabilization exercises alleviate pain and enhance functionality, in-person-supervised exercises may be more effective in improving functionality and reducing kinesiophobia in CLBP

CBMT, clinic-based McKenzie therapy; CPT, conventional physical therapy; CSS, current symptom score; CUA, cost utility analysis; DPT, digital application physical therapy; EARS, exercise adherence rating scale; FMS, functional movement screen; FTST, 5 times sit to stand test; GAD-7, generalised anxiety disorder-7; GROC, global rating of change; HEP, home exercise program; ICER, incremental cost-effectiveness ratio; IPAQ, international physical activity questionnaire; IPS, in-person supervised; LE, lower extremity; NRS, numeric rating scale; ODI, Oswestry disability index; PBQ, pain belief questionnaire; PCS, pain catastrophizing scale; PHQ, patient health questionnaire; PNE, pain neuroscience education; POMS, profile of mood states; PSEQ, pain self-efficacy questionnaire; PT, physiotherapist; QALY, quality adjusted life years; QBPDS, Quebec back pain disability scale; RMDQ, Roland Morris disability questionnaire; SDS, self-rating depression scale; SF, short form; SQI, sleep quality index; TBMT, telerehabilitation-based McKenzie therapy; TS, Tele-supervised; TSK, Tampa scale of kinesiophobia; US, unsupervised.

**Table 4:** Mobile health intervention features in the included studies.

Study ID	mHealth intervention	Features
Chhabra et al. 2018^8^	Mobile application “snapcare”	Personalized care in activity goals and home exercise program based on patient's baseline health data and pain levels after each activity session Goal advancement based on patient's comfort level with physical activity and exercise at various intervals, gauged via patient-reported outcome measures (PROMs) and exercise data collected via the app Addressed the improvements in physical activity, function, engagement, and compliance
Mbada et al. 2019^29^	Mobile application (TBMT) of mechanical diagnosis and therapy (MDT; McKenzie principles)	Personalized and guided self-therapy using the same protocols in the McKenzie protocol (ie, extension lying prone, extension in prone, and extension in standing) Exercises proceeded by back hygiene instructions. The app has features that allow users to pause, revert, or proceed to the next exercise. The app total run time is approximately 5 minutes Telemonitoring of adherence and utilization tracking of TBMT app—through phone calls and SMS to participants
Almhdawi et al. 2020^2^	Mobile application “relieve my back”	General advice and evidence-based instructions, office-based stretching exercises, and home-based strengthening exercises for lower back and abdominal muscles Four phone notifications (sound and vibration along with instruction pop-up screen) through the day to notify the participants to take a walk break, a reminder of the right posture, a reminder of the stretching exercises, and a reminder of the home-based exercises in the evening
Fatoye et al. 2020^13^	Mobile application (TBMT) of mechanical diagnosis and therapy (MDT; McKenzie principles)	Combination of McKenzie extension protocol and back care education Personalized and guided self-therapy using the same protocol as McKenzie protocol (ie, extension lying prone, extension in prone, and extension in standing) Telemonitoring of performance feedback and progress tracking; enhanced caregiver support to improve patient engagement and therapy compliance
Ozden et al. 2022^33^	Web-based telerehabilitation software Fizyoweb	User-friendly interface and detailed instructions for videos Features of monitoring the online activity of the patients and presenting statistical data to the clinician Messaging platform to allow communication with the clinician
Karaduman et al. 2023^24^	Videoconference	Stabilization exercise protocol, under the guidance of a physiotherapist Telephone calls from the physiotherapist at the end of weeks 1, 2 and 3, to provide feedback and support
Okudan et al. 2023^32^	Videoconference through Google Meets	Mobility, movement control, strengthening exercises, and functional exercises for patients with LBP, targeting the lumbar region, abdominal area, lower extremities, hips, and vertebral column PNE a) educational strategies to transfer information to the patients, via photographs, sharing analogies and stories, and explaining scientific research and its results b) Definitions related to pain, its transmission, physiology, and the representation of pain areas in the brain; different types of pain; concepts such as widespread pain and central and peripheral sensitization Patient engagement and active involvement in the conversation, sharing of thoughts and experiences

LBP, low back pain; PNE, pain neuroscience education.

#### 3.1.1. Risk of bias and publication bias

The risk of bias assessment across all outcomes revealed a mixed quality of studies. The overall risk of bias across the analysed outcomes from the included studies was generally “some concerns” to “high risk” (Figs. 2-15).

**Figure 2.**
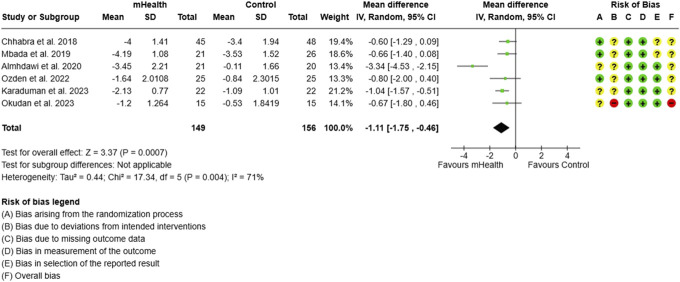
Forest plot and RoB for overall pain intensity. RoB, risk-of-bias.

**Figure 3.**
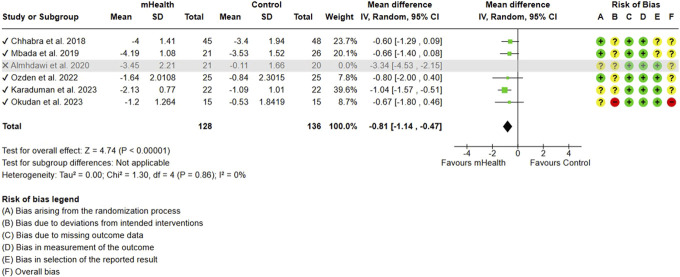
Sensitivity analysis for pain.

**Figure 4.**
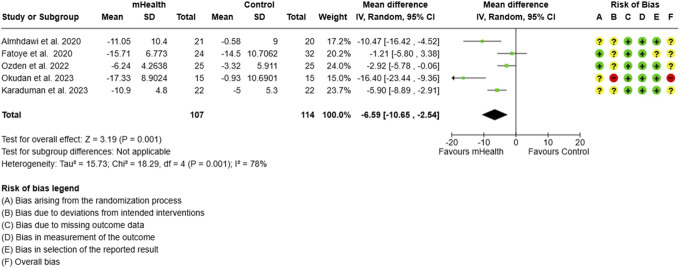
Forest plot and RoB for ODI. ODI, Oswestry disability index; RoB, risk-of-bias.

**Figure 5.**
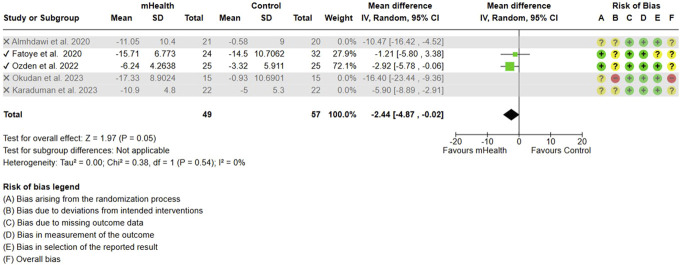
Sensitivity analysis for ODI. ODI, Oswestry disability index.

**Figure 6.**
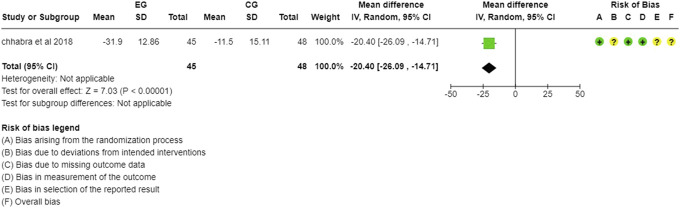
RoB for MODI. ODI, Oswestry disability index; RoB, risk-of-bias.

**Figure 7.**
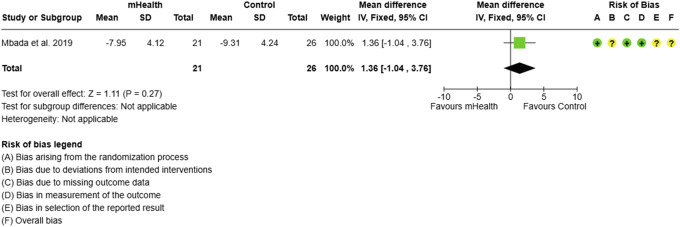
RoB for RMDQ. RMDQ, Roland Morris disability questionnaire; RoB, risk-of-bias.

**Figure 8.**
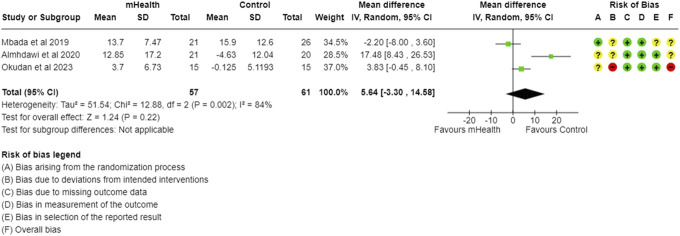
Forest plot and RoB for PCS (SF-12). PCS, physical component summary; RoB, risk-of-bias; SF, short form.

**Figure 9.**
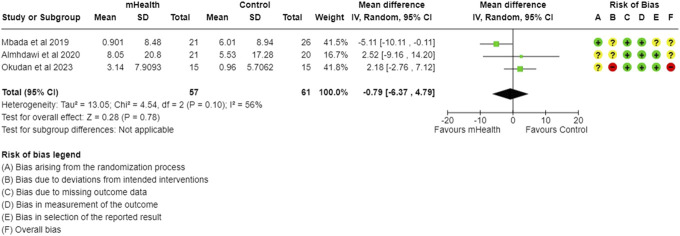
Forest plot and RoB for MCS (SF-12). MCS, mental component summary; RoB, risk-of-bias; SF, short form.

**Figure 10.**
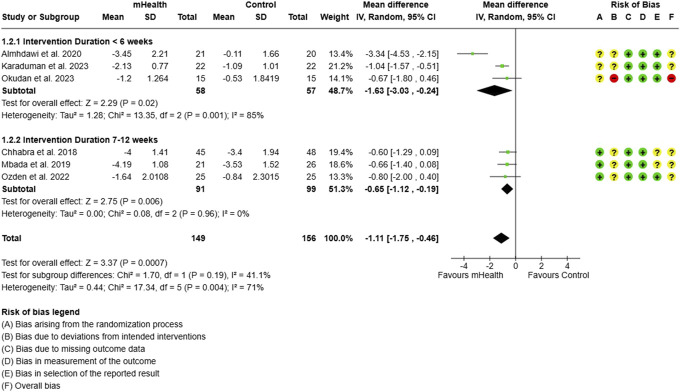
Subgroup analysis for pain: intervention duration.

**Figure 11.**
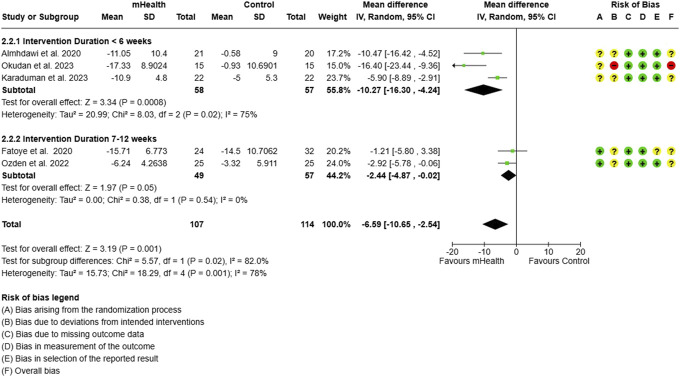
Subgroup analysis for ODI: intervention duration. ODI, Oswestry disability index.

**Figure 12.**
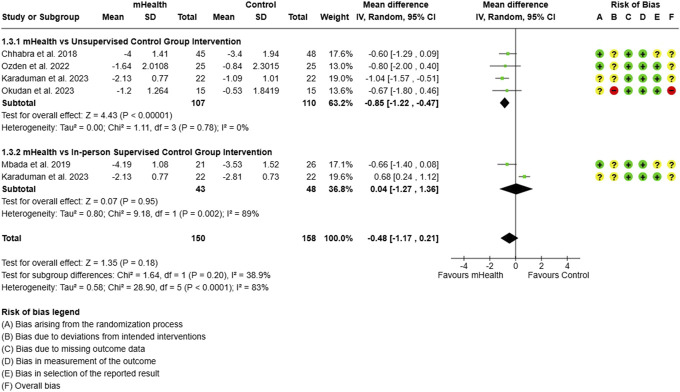
Subgroup analysis for pain: type of control group intervention.

**Figure 13.**
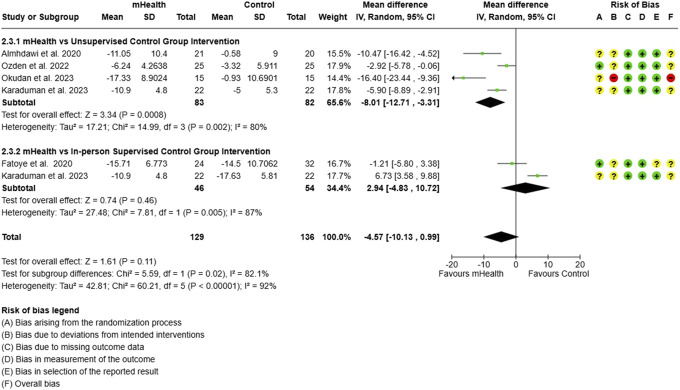
Subgroup analysis for ODI: type of control group intervention. ODI, Oswestry disability index.

**Figure 14.**
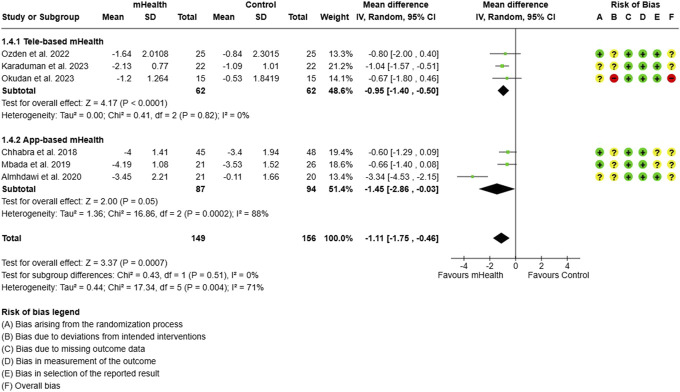
Subgroup analysis for pain: type of mHealth intervention.

**Figure 15.**
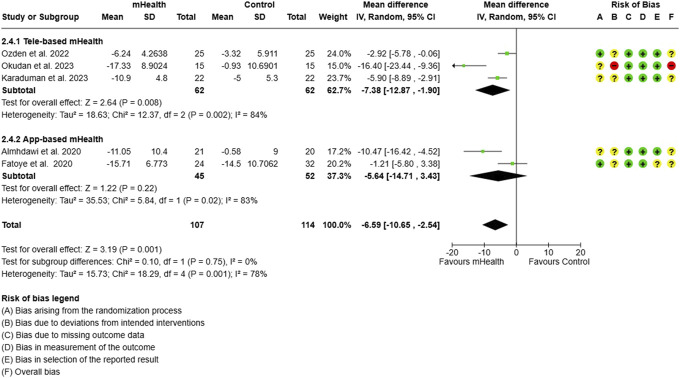
Subgroup analysis for ODI: type of mHealth intervention. ODI, Oswestry disability index.

For pain outcome, out of 6 studies, 3 had a low risk of bias,^8,29,33^ while rest had some concerns^1,24,32^ in the randomization process. One study^32^ was found to have high risk of bias due to intended interventions, while others had some concerns. Most studies had low risk of bias in 3 other domains. The Oswestry disability index (ODI) assessments showed a comparable distribution, with 2 low risk studies,^13,33^ and rest with some concerns for randomization. The risk of bias in ODI scores from other domains were comparable to that of pain outcome. Short form (SF-12) outcomes had fewer studies, but maintained a mix of low and some concerns ratings, with 1 study^32^ having high risk of bias due to deviation from intended interventions. The Roland Morris disability questionnaire (RMDQ) and MODI outcomes, while having fewer studies overall, also displayed a range of risk levels across different domains. Notably, the measurement of the outcome, missing outcome data, and selection of the reported result domains tended to have more low-risk ratings across all outcomes, suggesting stronger methodological quality in these areas. However, the presence of high risk and some concerns ratings in several studies and domains indicates that the overall evidence should be interpreted with caution, considering the potential impact of these methodological limitations on the reliability of the results.

Owing to limited number of studies (<10), the publication bias was not assessed.

#### 3.1.2. Pain intensity

The meta-analysis of 6 studies evaluating mHealth interventions, with 305 subjects, showed a significant reduction in the overall pain scores among participants using mobile health (mHealth) interventions compared to control groups. The pooled effect size indicated a significant mean difference (MD) of −1.11 (95% CI: −1.75 to −0.46), demonstrating the efficacy of mHealth interventions in alleviating CLBP in LMICs (Fig. 2). The included studies had a significantly high heterogeneity with an I^2^ of 71%, which was effectively reduced to 0% with a single study omission,^1^ decreasing the effect size to −0.81 (95% CI: −1.14 to −0.47) (Fig. 3).

#### 3.1.3. Disability

##### 3.1.3.1. Oswestry disability index

The meta-analysis including 5 studies evaluating mHealth interventions on 221 subjects showed a significant change in the subjects' functional disability measured by ODI scores. Due to lack of specific data in form of mean and SD, 1 study^13^ was not included in meta-analysis. The pooled analysis showed an MD of −6.59 (95% CI: −10.65 to −2.54), indicating a substantial reduction in disability levels among participants receiving mHealth interventions compared to controls (Fig. 4). Significant heterogeneity (I^2^ = 78%) was detected among the included studies. After excluding 3 studies^1,24,32^ using combinatorial algorithm, the heterogeneity was reduced to 0%, however, decreasing the effect size to −2.44 (Fig. 5).

##### 3.1.3.2. Modified Oswestry disability index

The modified Oswestry disability index (MODI) was evaluated by only 1 study; hence, the analysis could not be done. The authors reported a significant difference in the change in MODI scores across the 2 groups, favouring mHealth intervention over the routine care.^8^ Figure 6 shows the risk of bias assessment for MODI.

##### 3.1.3.3. Roland Morris disability questionnaire

The RMDQ was assessed by only 1 study, hence, could not be meta-analysed. The authors reported a significant difference in the change in RMDQ scores across the 2 groups, favouring mHealth intervention over the routine care.^29^ Figure 7 shows the risk of bias assessment for RMDQ.

#### 3.1.4. Short form-12 health survey

The SF-12 survey assesses the quality of life experienced by CLBP subjects. The data from 3 studies evaluating mHealth interventions across 118 CLBP participants revealed no improvements in both the physical component summary (PCS) and mental component summary (MCS) scores. The PCS scores showed an MD of 5.64 (95% CI: −3.30 to 14.58), while the MCS scores had an MD of −0.79 (95% CI: −6.37 to 4.79). The statistical heterogeneity was found to be high for PCS (I^2^ = 84%) and moderate for MCS (I^2^ = 56%) (Figs. 8 and 9).

Various subgroup analyses for primary outcomes were conducted to explore the potential sources of heterogeneity:

(1) Intervention Duration

Both short-term (lasting less than 6 weeks) and longer duration interventions (lasting 7–12 weeks) were found significantly effective in reducing pain in CLBP subjects. The effect size was, however, greater for shorter intervention durations. A meta-analysis of 3 studies,^1,24,32^ involving 115 CLBP subjects, showed that short-term mHealth interventions led to a substantial reduction in pain (MD = −1.63, 95% CI = −3.03, −0.24) (Fig. 10). In contrast, analysis of 3 studies^8,29,33^ with 190 subjects who received longer interventions also showed significant pain reduction, but with a smaller effect size (MD = −0.65, 95% CI: −1.12 to −0.19).

For ODI scores, the longer duration of the intervention produced a significant change (MD = −2.44; 95% CI = −4.87, −0.02) in disability across 106 subjects from 2 studies.^13,33^ The short-term interventions evaluated by 3 studies^1,24,32^ were, however, found to be significantly more effective in improving the functional disability (MD = −10.27, [95% CI = −16.30 to −4.24]) (Fig. 11).

(2) Type of Control Group

The efficacy of mHealth interventions was compared against different control conditions. The data from 217 participants evaluating mHealth interventions compared to unsupervised or home program for CLBP showed a significant reduction in pain (MD = −0.85, 95% CI: −1.22 to −0.47), while those compared to in-person supervised or clinic-based program demonstrated an insignificant effect on reduction of pain severity (0.04, 95% CI: −1.27 to 1.36) (Fig. 12).

Similar to the findings for pain reduction, the ODI scores were significantly reduced by mHealth interventions only when compared to an unsupervised program (MD = −8.01, 95% CI: −12.71 to −3.31). However, no significant difference was observed when mHealth interventions were compared to in-person supervised programs (Fig. 13).

(3) Type of mHealth intervention

Different types of mHealth interventions were also analysed. Mobile applications were found to be particularly effective in reducing pain (MD = −1.45, 95% CI: −2.86 to −0.03) by 3 studies on 181 CLBP subjects, whereas tele-based interventions in 3 studies on 124 CLBP subjects showed a slightly lesser, however, significant effect (−0.95, 95% CI: −1.40 to −0.50) (Fig. 14).

The ODI scores showed a significant reduction with the use of tele-based mHealth interventions against the control group care (MD = −7.38; 95% CI = −12.87 to −1.90). App-based interventions, however, did not produce any difference for the functional disability in CLBP subjects (Fig. 15).

#### 3.1.5. Certainty of evidence

The evidence varied in certainty across different outcomes, ranging from very low to moderate (Supplementary file 2, http://links.lww.com/PR9/A281). Notably, pain outcome was rated as having “moderate” certainty of evidence. Functional disability showed mixed results: when assessed with ODI, the certainty was low; with MODI and RMDQ, it was moderate. For both components of SF-12 (PCS, MCS), the certainty of evidence was low and very low. The main factors affecting certainty were risk of bias in the studies, with some outcomes showing serious or very serious concerns. Publication bias was strongly suspected for the ODI functional disability outcome, leading to its downgrading by 1 level. In addition, inconsistency was noted for the PCS outcome, which was also, therefore, downgraded by 1 level. Overall, the evidence ranged from very low to moderate certainty, suggesting that further research could potentially affect the confidence in these results.

#### 3.1.6. Summary of findings

The mobile health intervention for CLBP in LMICs demonstrated mixed results with varying levels of certainty. With moderate certainty, the intervention likely produces slight reduction in pain intensity. However, it reduces functional disability as measured by ODI, but not RMDQ. The effect on the both physical and mental components of quality of life remains very uncertain. Overall, while the mobile health intervention shows some promise in specific areas, its benefits appear limited across most outcomes (Supplementary file 3, http://links.lww.com/PR9/A281).

#### 3.1.7. Trial sequential analysis

For both pain and ODI, the last point on the cumulative z-score line crossed the conventional test boundary, but not the monitoring boundaries, suggesting a statistical difference in the conventional meta-analysis but not in TSA (Supplementary file 4, 5, http://links.lww.com/PR9/A281). The z-curve for both outcomes reached the RIS and remained above the futility boundary; however, it did not cross the TSA monitoring boundary for benefit, suggesting that additional studies may be required to determine a more conclusive effect.^23^

We did not conduct TSA for secondary outcomes due to insufficient data.

## 4. Discussion

By critically evaluating the available evidence, this SRMA aimed to provide comprehensive insights into the effectiveness of mHealth interventions in managing CLBP within LMICs. The findings of this SRMA from 7 included studies highlight both the potential benefits and the challenges associated with implementing mHealth technologies in resource-constrained settings. The effectiveness of mHealth interventions for pain reduction in LMICs is particularly significant given the challenges these countries face in health care delivery. As highlighted in the Global Burden of Disease Study, LMICs bear a disproportionate burden of CLBP.^17^ Our findings suggest that mHealth interventions could play a crucial role in addressing this burden by providing accessible and effective pain management solutions.

The implementation of mHealth interventions for self-management exemplifies a significant disparity between HICs and their low- and middle-income counterparts.^20^ HICs benefit from widespread smartphone availability, robust cellular networks, and high-speed internet, enabling sophisticated mHealth solutions like real-time pain monitoring apps, telehealth consultations, and personalized digital exercise programs. In HICs, the adoption of established mHealth initiatives as well as the use of mobile technologies for patient records has been far more in proportion to that in LMICs, as per WHO Global Observatory for eHealth.^12^ It is, therefore, important to consider the challenges of implementing mHealth interventions in LMICs.^6^ Low- and middle-income countries face significant challenges in leveraging mHealth technologies for LBP management. Limited smartphone access, unreliable internet connectivity, and lower technological infrastructure and expertise impede the widespread adoption of mHealth solutions in LMICs, in addition to the cultural and language barriers.^12^ While mHealth has the potential to improve back pain care globally, its current implementation widens the health care gap between HICs and LMICs. Given the abundance of mHealth studies predominantly from the HICs, it, therefore, becomes imperative to comprehensively review the existing literature exploring the effectiveness of mHealth in LBP care in the context of LMICs alone.

### 4.1. Pain intensity

Our meta-analysis revealed a significant reduction in overall pain scores among participants using mHealth interventions compared to control groups (MD −1.11; 95% CI: −1.75 to −0.46); however, this result is likely more influenced by the effect of mHealth interventions relative to unsupervised interventions. Our result is comparable to the previous 2 systematic reviews, which reported a MD of −0.85^7^ and −0.86,^21^ respectively, favouring mHealth for the reduction of pain. The softwares that delivered exercise intervention alone and in combination with other interventions had a significantly greater impact on pain intensity.^21^

Subgroup analyses investigating potential effect modifiers like intervention type and duration revealed interesting patterns. Both short (under 6 weeks) and longer (7–12 weeks) interventions were significantly effective in reducing pain, with shorter interventions showing a slightly greater effect size, indicating a potentially better impact of mHealth technologies on pain relief. This is especially advantageous in resource-limited settings where prolonged interventions may be challenging to implement. However, with only few studies per subgroup, these duration comparisons for pain relief should be interpreted cautiously. Unlike our findings, Dario et al. (2017)^10^ reviewed 11 telehealth studies on patients with CLBP and found no significant effect on pain reduction for either short- or long-term interventions. This discrepancy may stem from their inclusion of LBP subjects regardless of pain duration and distinct operational definition of short- and long-term interventions.

Our review additionally found that mHealth interventions are significantly more effective in reducing pain compared to unsupervised or home-based programs, although not significantly different from in-person supervised programs. This highlights the potential of mHealth as a viable and superior alternative to unsupervised care, potentially addressing issues of accessibility and continuity of care in LMICs. The same trend was observed for the ODI scores. Integrating audio narration with video practice enhances learning efficiency by combining visual and auditory stimuli, resulting in better comprehension and retention than traditional on-paper infographics.^22^ This integrated approach provides a more engaging learning experience, maximizing the benefits of both visual and auditory inputs. Our findings align with a prior systematic review showing that technology-supported exercise therapy is as effective as traditional physical therapy for chronic pain, including CLBP.^3^ In addition, past studies have shown that supervised exercise programs are generally more effective than unsupervised ones for managing CLBP.^28^ Long-term adherence to home-based programs is crucial for sustained improvement, with patients who consistently follow prescribed exercises seeing notable gains in physical function.^28^ Our results are also partially consistent with previous studies, indicating that telerehabilitation is comparable to in-person care and more effective than home-based or no intervention.^26,38,43^

Regarding the mode of mHealth delivery, the mobile applications showed significantly greater pain reduction than the control group, relative to the tele-based interventions. This difference could be attributed to the accessibility and convenience of mobile apps, which allow for more frequent and consistent engagement with the intervention.^27^ Furthermore, the applications provide timely notifications to the participants, which likely provides extra motivation to adhere to their management.

The greater effectiveness of mHealth intervention over routine care in managing CLBP can be attributed to its ability to enhance patient care through remote monitoring and promoting better adherence and self-care in patients' own environments. Muñoz-Tomás et al.^30^ (2023) highlighted that real-time communication via telerehabilitation allows physiotherapists to observe and guide patients, ensuring proper execution of exercises. Fernandes et al.^16^ (2022) demonstrated that mHealth intervention, delivered through mobile applications, videoconferencing platforms, or websites, is an effective and satisfactory approach for managing musculoskeletal problems. This aligns well with our positive outcomes for mHealth interventions, further supporting their potential to improve pain management in resource-limited settings, as observed in our study.

However, it is important to note that our review specifically focused on LMICs, whereas most previous reviews have predominantly included studies from HICs.^21,31^ This distinction is crucial, as the content and, thereby, the effectiveness of mHealth interventions may vary across different socioeconomic contexts. Our findings suggest that the benefits of mHealth for pain reduction observed in HICs may also extend to LMIC settings, which is promising for addressing the global burden of CLBP.

### 4.2. Functional disability

The analysis of functional disability, as measured by the ODI, showed a significant difference in reduction in disability levels among participants receiving mHealth interventions compared to controls. However, this effect is likely more influenced by the impact of mHealth interventions relative to unsupervised care, similar to the observed outcomes for pain. These findings partially corroborate with some previous reviews. Scholz et al.^36^ (2023) found a small to medium effect favouring the intervention group (with a pooled summary effect size of Hedges g = 0.43, 95% CI = 0.27–0.59). Their meta-analysis combined the studies that used various pain disability assessment tools (such as RMDQ, ODI, and MODI). This approach makes it challenging to interpret the precise results due to the distinct content and scoring methods of each scale. In addition, most of the included studies were conducted in HICs, thus rendering it difficult to compare their results with ours. Another review by Hong et al.^21^ (2024) reported a significant MD of −8.21 (95% CI = −13.02 to −3.39) in ODI, while insignificant for RMDQ (MD = −2.03; 95% CI = −4.62 to 0.56). Similar findings were reported by Shizheng Du et al.^11^ (2017), who found a significant effect in reducing disability using self-management programs in LBP subjects. The discrepancy in disability outcomes between our review and previous studies could be attributed to several factors: differences in intervention design and content tailored for LMIC settings, variation in health care systems and access to complementary care, and the socioeconomic factors that may influence the translation of pain reduction to functional improvements. These differences highlight the need for further research specifically designed for and conducted in LMIC contexts to better understand the impact of mHealth interventions on functional disability in these settings.

For ODI, the longer duration interventions (7–12 weeks) showed lesser improvement in disability, relative to short-term interventions. This suggests that longer-duration mHealth interventions may not be necessary to achieve meaningful improvements in functional outcomes. Lara-Palomo et al.^26^ (2022) compared the 2 groups for change in pain and functional status with respect to intervention duration. They, however, reported no significant difference in pain or back-specific functional status between mHealth and control groups, for both short-duration (4–6 weeks) and long-duration (3–6 months) interventions.

The network meta-analysis conducted by Slattery et al.^39^ (2019) suggested that the mobile app–based interventions have a 43% probability of being the most effective in reducing pain interference or pain-related disability in subjects with chronic pain, as against 0% for the videoconferencing-based interventions. In contrast, the ODI scores in our review responded better with the tele-based interventions. However, as these results are based on only 2 to 3 studies per subgroup, they should be interpreted with caution.

### 4.3. Quality of life

The analysis of SF-12 survey results showed nonsignificant improvements in both physical and mental components by mHealth intervention, over the control group. This suggests that the impact of mHealth interventions on overall quality of life may be limited or require longer intervention periods to manifest measurable changes. This finding is, however, somewhat inconsistent with previous reviews that have reported small but significant improvements in quality of life with digital interventions for CLBP.^10^ However, it is worth noting that quality of life outcomes can be influenced by various factors beyond pain and disability, including cultural and socioeconomic variables that may be particularly relevant in LMIC contexts.

Our findings suggest that while mHealth interventions may effectively reduce pain more than the control group intervention, their impact on disability and quality of life is less clear and warrants further investigation. This finding is in line with a previous study,^10^ which reported mixed results on the impact of mHealth on functional outcomes in patients with CLBP.

Despite the positive outcomes, significant heterogeneity was observed in the included studies, suggesting variability in intervention types and durations, study populations, and settings. However, even after the adjustments done through sensitivity analysis, the effect estimates were reduced markedly, reflecting the need for more standardized approaches in future research to reduce variability. Also, most of the studies included were found to have “some concerns,” while one had a high risk of bias primarily due to deviation from the intended intervention, thus compromising the quality of studies. The TSA for the primary outcomes additionally suggested the need for further trials in LMIC settings, as the cumulative z-score line for either of the outcomes did not cross the TSA monitoring boundary for benefit.

### 4.4. Strengths, limitations, and future directions

To the best of the authors' knowledge, this is the first SRMA to comprehensively evaluate the impact of mHealth interventions in improving pain and functions in patients with CLBP in LMICs. The strength of this SRMA lies in its systematic and robust methodology to identify, collate, critically appraise, and synthesize the evidence informing the review topic. Two reviewers with an audit and arbitration process were adopted for screening, appraisal, synthesis, and grading certainty of evidence.

This SRMA has a few limitations. The included studies varied widely in their methodological quality, intervention types, and outcome measures, contributing to the observed heterogeneity. In addition, the reliance on self-reported pain and disability measures may introduce bias. The overall risk of bias across the analyzed outcomes was generally “some concerns” to “high risk,” which may affect the reliability of the results. Given the importance of context in this SRMA, the exclusion of grey literature and non-English studies might have led to missing relevant articles. However, searching of multiple subject-specific and multidisciplinary databases along with reference list searching until data saturation (defined as no new articles identified) were adopted to ensure that the best available evidence informed the review findings. Another limitation of our review is the classification of the 1 study^29^ as “quasi-experimental” by the authors, despite its meeting key criteria of RCT, such as random allocation with a permuted block method and inclusion of a CONSORT flow diagram.

Future research should aim to standardize the intervention protocols and outcome measures to enhance comparability across studies. Furthermore, larger and higher quality RCTs are needed to confirm these findings and explore the long-term sustainability of mHealth interventions in managing CLBP. Also, the views and attitudes of patients should be systematically studied to explore patients' acceptance of and challenges faced in using mHealth interventions.

## 5. Conclusion

This review provides evidence that mHealth interventions is potentially effective in reducing pain and functional disability associated with CLBP in LMICs, particularly when compared to unsupervised care. However, their impact on quality of life remains inconclusive. The findings highlight the potential of mHealth as an accessible and scalable approach to CLBP management in resource-constrained settings, while also underscoring the need for further research to strengthen the evidence base and optimize intervention strategies.

## Disclosures

The authors have no conflict of interest to declare.

## Supplemental audio content

Supplemental digital content associated with this article can be found online at http://links.lww.com/PR9/A281.
