# Supplementary material for: Efficacy of mobile health interventions in the conservative management of chronic low back pain in low- and middle-income countries: a systematic review, meta-analysis, and trial sequential analysis
Source: Pain Rep. 2025 Feb 13;10(2):e1242. doi: 10.1097/PR9.0000000000001242 (PMC11826050; doi:10.1097/PR9.0000000000001242)
Supplement: Supplementary file 1 [file painreports-10-e1242-s001.pdf]

Supplementary File 1. Database search strategy

Database	Search Strategy	Number of articles
PubMed	((low back pain OR chronic low back pain OR lumbago OR lumbar radiculopathy) AND ((mobile health OR mHealth OR telehealth OR telerehabilitation OR telemedicine) OR (mobile application OR telemedicine OR text messaging OR mobile phone OR smartphone OR social media OR internet)))<br><br>("low back pain"[MeSH Terms] OR ("low"[All Fields] AND "back"[All Fields] AND "pain"[All Fields]) OR "low back pain"[All Fields] OR (("chronic"[All Fields] OR "chronical"[All Fields] OR "chronically"[All Fields] OR "chronicities"[All Fields] OR "chronicity"[All Fields] OR "chronicization"[All Fields] OR "chronics"[All Fields]) AND ("low back pain"[MeSH Terms] OR ("low"[All Fields] AND "back"[All Fields] AND "pain"[All Fields]) OR "low back pain"[All Fields])) OR ("low back pain"[MeSH Terms] OR ("low"[All Fields] AND "back"[All Fields] AND "pain"[All Fields]) OR "low back pain"[All Fields] OR "lumbago"[All Fields]) OR (("lumbarised"[All Fields] OR "lumbarization"[All Fields] OR "lumbarized"[All Fields] OR "lumbars"[All Fields] OR "lumbosacral region"[MeSH Terms] OR ("lumbosacral"[All Fields] AND "region"[All Fields]) OR "lumbosacral region"[All Fields] OR "lumbar"[All Fields]) AND ("radiculopathy"[MeSH Terms] OR "radiculopathy"[All Fields] OR "radiculopathies"[All Fields]))) AND ("telemedicine"[MeSH Terms] OR "telemedicine"[All Fields] OR ("mobile"[All Fields] AND "health"[All Fields]) OR "mobile health"[All Fields] OR ("mhealth s"[All Fields] OR "telemedicine"[MeSH Terms] OR "telemedicine"[All Fields] OR "mhealth"[All Fields]) OR ("telehealth s"[All Fields] OR "telemedicine"[MeSH Terms] OR "telemedicine"[All Fields] OR "telehealth"[All Fields]) OR ("telerehabilitation"[MeSH Terms] OR "telerehabilitation"[All Fields]) OR ("telemedicine"[MeSH Terms] OR "telemedicine"[All Fields] OR "telemedicine s"[All Fields]) OR ("mobile applications"[MeSH Terms] OR ("mobile"[All Fields] AND "applications"[All Fields]) OR "mobile applications"[All Fields] OR ("mobile"[All Fields] AND "application"[All Fields]) OR "mobile application"[All Fields] OR ("telemedicine"[MeSH Terms] OR "telemedicine"[All Fields] OR "telemedicine s"[All Fields]) OR ("text messaging"[MeSH Terms] OR ("text"[All Fields] AND "messaging"[All Fields]) OR "text messaging"[All Fields]) OR ("cell phone"[MeSH Terms] OR ("cell"[All Fields] AND "phone"[All Fields]) OR "cell phone"[All Fields] OR ("mobile"[All Fields] AND "phone"[All Fields]) OR "mobile phone"[All Fields]) OR ("smartphone"[MeSH Terms] OR "smartphone"[All Fields] OR "smartphones"[All Fields] OR "smartphone s"[All Fields]) OR ("social media"[MeSH Terms] OR ("social"[All Fields] AND "media"[All Fields]) OR "social media"[All Fields]) OR ("internet"[MeSH Terms] OR "internet"[All Fields] OR "internet s"[All Fields] OR "internets"[All Fields])))	729
Scopus	(mobile AND health OR mhealth OR telehealth OR telerehabilitation OR telemedicine) AND (mobile AND application OR text AND messaging OR mobile AND phone OR smartphone OR social AND	47

	media OR internet) AND (low AND back AND pain OR chronic AND low AND back AND pain OR lumbago OR lumbar AND radiculopathy)	
WoS	(ALL=((mobile health OR mHealth OR telehealth OR telerehabilitation OR telemedicine) OR (mobile application OR text messaging OR mobile phone OR smartphone OR social media OR internet))) AND ALL=(low back pain OR chronic low back pain OR lumbago OR lumbar radiculopathy)	1046
EMBASE	(**mobile** AND **health** OR **mhealth** OR **telehealth** OR **telerehabilitation** OR **telemedicine**) AND (**mobile** AND **application** OR (**text** AND **messaging**) OR (**mobile** AND **phone**) OR **smartphone** OR (**social** AND **media**) OR **internet**) AND (**'low back pain'**/exp OR **'low back pain'** OR (**low** AND (**'back'**/exp OR **back**) AND (**'pain'**/exp OR **pain**)) OR **'chronic low back pain'**/exp OR **'chronic low back pain'** OR (**chronic** AND **low** AND (**'back'**/exp OR **back**) AND (**'pain'**/exp OR **pain**)) OR **'lumbago'**/exp OR **lumbago** OR **'lumbar radiculopathy'**/exp OR **'lumbar radiculopathy'**)	243
CINAHL	(mobile health OR mhealth OR telehealth OR telerehabilitation OR telemedicine) AND (mobile application OR text messaging OR mobile phone OR smartphone OR social media OR internet) AND (low back pain OR chronic low back pain OR lumbago OR lumbar radiculopathy)	262

Author(s):
Question: Mobile Health intervention compared to Control intervention for CLBP
Setting: Low and Middle Income Countries
Bibliography: Rani B. Mobile health versus Conventional/in-person for CLBP. Cochrane Database of Systematic Reviews [Year], Issue [Issue].

Certainty assessment							№ of patients		Effect		Certainty	Importance
**№ of studies**	**Study design**	**Risk of bias**	**Inconsistency**	**Indirectness**	**Imprecision**	**Other considerations**	**mobile Health intervention**	**Control intervention**	**Relative (95% CI)**	**Absolute (95% CI)**		
**Pain intensity (assessed with: VAS/NPRS)**												
6	randomised trials	serious[a]	not serious	not serious	not serious	none	149	156	-	MD **1.11 more** (1.75 fewer to 0.46 fewer)	⨁⨁⨁◯ Moderate	CRITICAL
**Functional Disability (assessed with: ODI)**												
5	randomised trials	serious[a]	not serious	not serious	not serious	publication bias strongly suspected[b]	107	114	-	MD **6.59 more** (10.65 fewer to 2.54 fewer)	⨁⨁◯◯ Low	CRITICAL
**Functional Disability (assessed with: MODI)**												
1	randomised trials	serious[a]	not serious	not serious	not serious	none	45	48	-	MD **20.4 more** (26.09 fewer to 14.71 fewer)	⨁⨁⨁◯ Moderate	CRITICAL
**Functional Disability (assessed with: RMDQ)**												
1	randomised trials	serious[a]	not serious	not serious	not serious	none	21	26	-	MD **1.36 higher** (1.04 lower to 3.76 higher)	⨁⨁⨁◯ Moderate	CRITICAL
**MCS (assessed with: SF-12)**												
3	randomised trials	very serious[c]	not serious	not serious	not serious	none	57	61	-	MD **0.79 more** (6.37 fewer to 4.79 more)	⨁⨁◯◯ Low	IMPORTANT
**PCS (assessed with: SF-12)**												
3	randomised trials	very serious[c]	serious[d]	not serious	not serious	none	57	61	-	MD **5.64 more** (3.3 fewer to 14.58 more)	⨁◯◯◯ Very low	IMPORTANT

CI: confidence interval; **MD:** mean difference

Explanations

a. 'some concerns' in Risk of bias
b. as per funnel plot
c. high risk of bias in 2 studies
d. different direction of results in two studies' control group

Supplementary file 2: GRADE evidence profile

Summary of findings:

Mobile Health intervention compared to Control intervention for CLBP

Patient or population: CLBP
Setting: Low and Middle Income Countries
Intervention: mobile Health intervention
Comparison: Control intervention

Outcomes	Anticipated absolute effects* (95% CI)		Relative effect (95% CI)	№ of participants (studies)	Certainty of the evidence (GRADE)	Comments
	Risk with Control intervention	Risk with mobile Health intervention				
Pain intensity assessed with: VAS/NPRS	The mean pain intensity was - **1.58**	MD **1.11 more** (1.75 fewer to 0.46 fewer)	-	305 (6 RCTs)	⊕⊕⊕◯ Moderate[a]	Mobile Health intervention likely results in a slight reduction in pain intensity.
Functional Disability assessed with: ODI	The mean functional Disability was - **4.86**	MD **6.59 more** (10.65 fewer to 2.54 fewer)	-	221 (5 RCTs)	⊕⊕◯◯ Low[a,b]	The evidence suggests mobile Health intervention reduces functional Disability.
Functional Disability assessed with: MODI	The mean functional Disability was - **11.5**	MD **20.4 more** (26.09 fewer to 14.71 fewer)	-	93 (1 RCT)	⊕⊕⊕◯ Moderate[a]	Mobile Health intervention likely reduces functional Disability.
Functional Disability assessed with: RMDQ	The mean functional Disability was - **9.31**	MD **1.36 higher** (1.04 lower to 3.76 higher)	-	47 (1 RCT)	⊕⊕⊕◯ Moderate[a]	Mobile Health intervention likely does not reduce RMDQ.
MCS assessed with: SF-12	The mean MCS was **3.365**	MD **0.79 more** (6.37 fewer to 4.79 more)	-	118 (3 RCTs)	⊕⊕◯◯ Low[c]	The evidence suggests that mobile Health intervention does not increase MCS.
PCS assessed with: SF-12	The mean PCS was **2.755**	MD **5.64 more** (3.3 fewer to 14.58 more)	-	118 (3 RCTs)	⊕◯◯◯ Very low[c,d]	The evidence is very uncertain about the effect of mobile Health intervention on PCS.

***The risk in the intervention group** (and its 95% confidence interval) is based on the assumed risk in the comparison group and the **relative effect** of the intervention (and its 95% CI).

CI: confidence interval; **MD:** mean difference

GRADE Working Group grades of evidence
High certainty: we are very confident that the true effect lies close to that of the estimate of the effect.
Moderate certainty: we are moderately confident in the effect estimate: the true effect is likely to be close to the estimate of the effect, but there is a possibility that it is substantially different.
Low certainty: our confidence in the effect estimate is limited: the true effect may be substantially different from the estimate of the effect.
Very low certainty: we have very little confidence in the effect estimate: the true effect is likely to be substantially different from the estimate of effect.

Explanations

a. 'some concerns' in Risk of bias
b. as per funnel plot
c. high risk of bias in 2 studies
d. different direction of results in two studies' control group

Supplementary file 3: Summary of findings

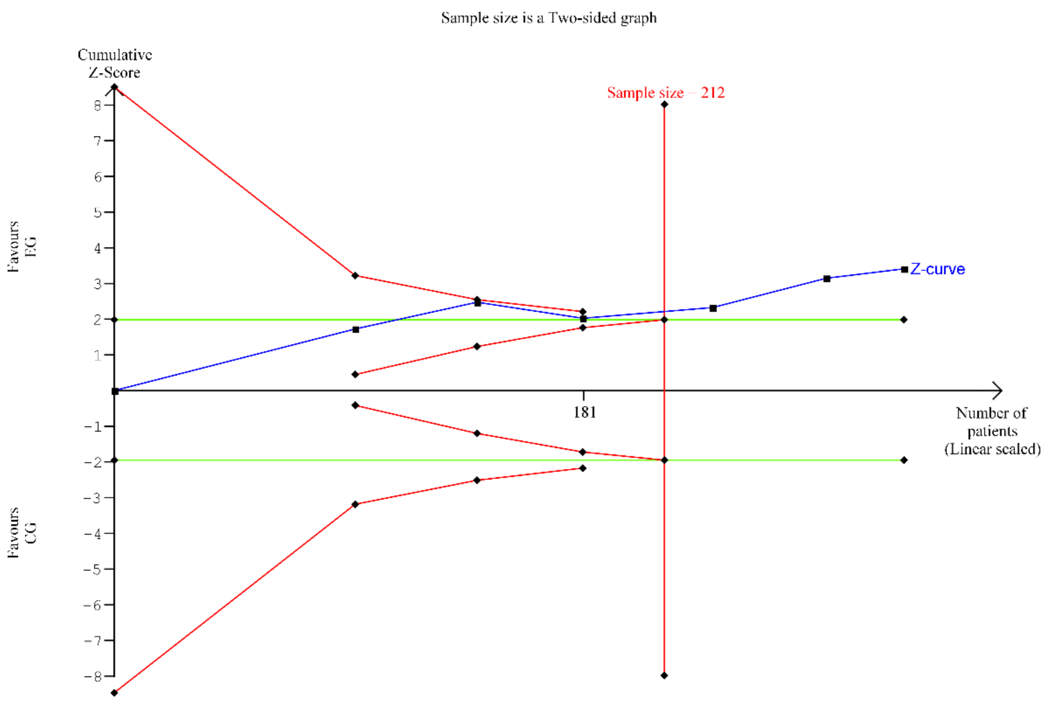

Supplementary file 4: Trial Sequential Analysis for pain

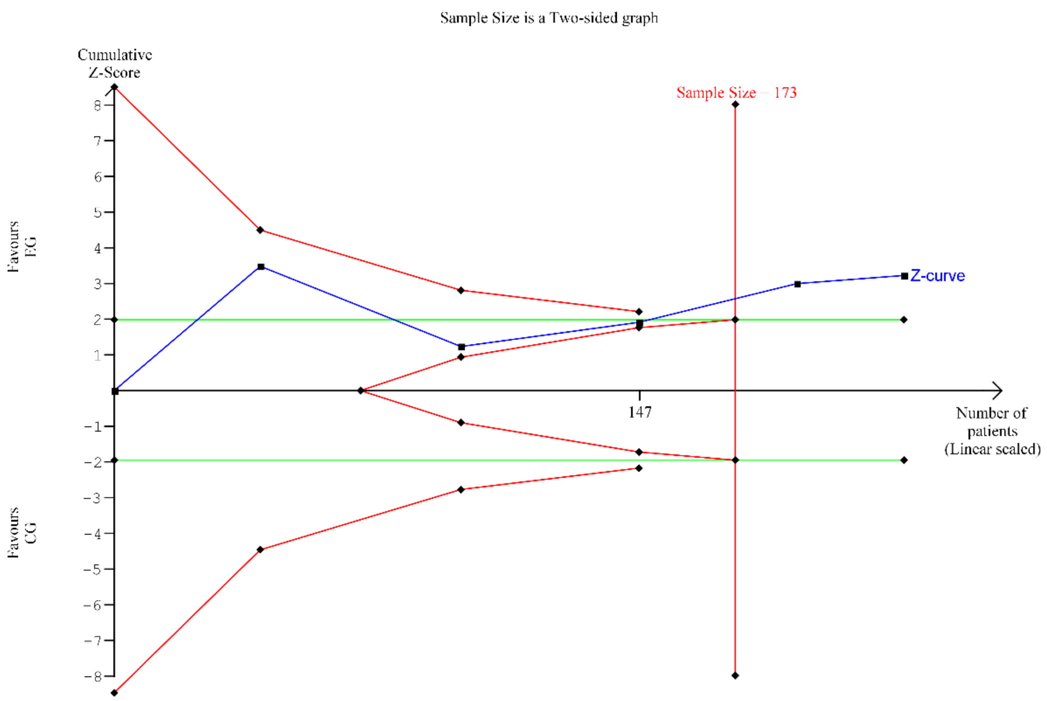

Supplementary file 5: Trial Sequential Analysis for ODI
